# Genetic Diversity and Zoonotic Potential of *Blastocystis* in Korean Water Deer, *Hydropotes inermis argyropus*

**DOI:** 10.3390/pathogens9110955

**Published:** 2020-11-17

**Authors:** Kyoo-Tae Kim, Gyeonguk Noh, Haeseung Lee, Seon-Hee Kim, Hyesung Jeong, Yongkwan Kim, Weon-Hwa Jheong, Jae-Ku Oem, Tae-Hwan Kim, Oh-Deog Kwon, Dongmi Kwak

**Affiliations:** 1 Animal Health Center of Zoo Land, Daejeon O-World, Daejeon 35073, Korea; zoovet@dcco.kr; 2 Department of Veterinary Medicine, College of Veterinary Medicine, Kyungpook National University, Daegu 41566, Korea; ku0207@naver.com (G.N.); lhspppp@naver.com (H.L.); thkim56@knu.ac.kr (T.-H.K.); odkwon@knu.ac.kr (O.-D.K.); 3 Environmental Health Research Department, National Institution of Environmental Research, Incheon 22689, Korea; sunny14@korea.kr (S.-H.K.); halley@korea.kr (H.J.); kyk5388@korea.kr (Y.K.); purify@korea.kr (W.-H.J.); 4 Department of Veterinary Medicine, College of Veterinary Medicine, Jeonbuk National University, Jeonbuk 54596, Korea; jku0623@jbnu.ac.kr; 5 Cardiovascular Research Institute, Kyungpook National University, Daegu 41944, Korea

**Keywords:** *Blastocystis*, *Hydropotes inermis argyropus*, Korean water deer, phylogeny, subtype, wildlife animal

## Abstract

*Blastocystis* is a protozoan parasite commonly detected in the intestinal tract of humans and animals. It has been actively studied worldwide; however, information on *Blastocystis* is limited in Korea. Because there is an increasing concern about the contact between wildlife and domestic animals or humans, we assessed the infection status and zoonotic potential of *Blastocystis* in Korean water deer (KWD, *Hydropotes inermis argyropus*) using genotyping and phylogenetic analysis. A total of 125 fresh fecal samples were collected from KWD which were killed by vehicles on highways or roadsides in this study. Among the 125 samples, 51 (40.8%) were PCR positive. We performed nucleotide sequencing and phylogenetic analysis of 26 of the 51 PCR-positive samples. By analyzing *Blastocystis* 18S rRNA, two subtypes (ST4 and ST14) were identified in this study. Of the 26 samples analyzed, 25 were identified as ST14 and one as ST4. Infection of ST14 in humans has not been reported. Although only one ST4 sample was detected in this study, ST4 has zoonotic potential without showing ruminant specificity. Thus, continuous attention should be provided to the potential of transmission between wildlife and domestic animals and humans.

## 1. Introduction

*Blastocystis* belongs to the stramenopiles, commonly found in the intestinal tract of humans and animals and has been associated with intestinal disorders. However, its pathogenicity remains controversial because many infected humans and animals are asymptomatic [1,2]. However, zoonotic potential is crucial because the infection rates of animal handlers, such as people working in laboratories and zoos, are much higher than those who do not [3]. Meanwhile, this parasite has broad genetic diversity [4]. At least 17 subtypes of *Blastocystis* exist, and most exhibit low host specificity, indicating a broad host range [5]. For these reasons, studies on *Blastocystis* are actively conducted in humans and domestic animals worldwide.

The Korean water deer (KWD, *Hydropotes inermis argyropus*) is a wild animal that has prominent tusks but does not have antlers. From 1982 to 2011, reforestation programs were implemented because of a significant loss of forests due to the Korean War in 1950–1953. During the reforestation programs, the population of KWD increased greatly [6]. Furthermore, the population of KWD greatly increased because no natural predators exist in Korea. They mark out their territory using urine and feces. As the population of KWD increases, KWD often appear on farms looking for food. Moreover, major transmission of *Blastocystis* is through the fecal-oral and water-borne routes. Therefore, *Blastocystis* infection in KWD is important in assessing its potential transmission to domestic animals and humans [7,8].

While studies were conducted on *Blastocystis* in domestic animals, including cattle and pigs, in Korea [9,10], little information is available on *Blastocystis* in wild animals, except for wild boar [11]. Thus, this study assesses the distribution and subtypes of *Blastocystis* in wild KWD and evaluates their zoonotic potential.

## 2. Results and Discussion

In this study, we collected 125 fecal samples from KWD and examined their infection status. Fifty-one (40.8%) of the 125 samples were positive of *Blastocystis* by PCR. While examining, we collected the following factors: sex, collected region, and season (Table 1).

While the pathogenicity of *Blastocystis* in causing intestinal disorders is controversial because many infected humans and animals are asymptomatic [1,2], studies on *Blastocystis* have been actively conducted due to its zoonotic potential and genetic diversity [4]. However, few *Blastocystis* studies on wild animals including KWD exist worldwide. Therefore, according to the available data, the infection rates of *Blastocystis* were compared among deer, red deer, reindeer, sika deer, mouse deer, roe deer, and fallow deer. The infection rate of *Blastocystis* in red deer (2%) in Australia was much lower than that in KWD [12]. Similarly, the infection rates of *Blastocystis* in reindeer (6.7%) and sika deer (14.6%) in China were lower than that in KWD [13]. However, the infection rates of *Blastocystis* in mouse deer (100%), roe deer (50%), and fallow deer (100%) in the United Kingdom and Mauritius were higher than that in KWD despite the small sample number [14].

Our study indicates that the correlation between *Blastocystis* infection rate and sex was statistically significant (*p* = 0.015) because the infection rate in males (38.2%) was higher than that in females (23.3%). However, the infection rate in unknown data (57.5%) was much higher than those in males and females. Therefore, a further study on the correlation between *Blastocystis* infection and sex is needed to clarify whether sex is a risk factor for *Blastocystis* infection.

According to the provincial boundaries in Korea, the infection rate in the central region was higher (47.8%) than those in the northern (41.6%) and southern regions (30.6%). However, statistical analysis indicated that infection rate was not significantly correlated with region (*p* = 0.346). However, the infection rate in unknown samples is higher than that in the central region; thus, a further study on the correlation between *Blastocystis* infection demonstrating a uniform distribution through the country is required.

The collection seasons of the samples were divided into two groups: spring (March to May) and summer (June to August). The infection rate in spring (43.8%) was higher than that in summer (34.9%). However, *Blastocystis* infection was not significantly correlated with season (*p* = 0.682). There were no data for fall and winter because road-killed or trapped KWD samples were not submitted. To clarify whether season is a risk factor for *Blastocystis* infection of KWD, a further study on season is warranted.

**Figure 1:**
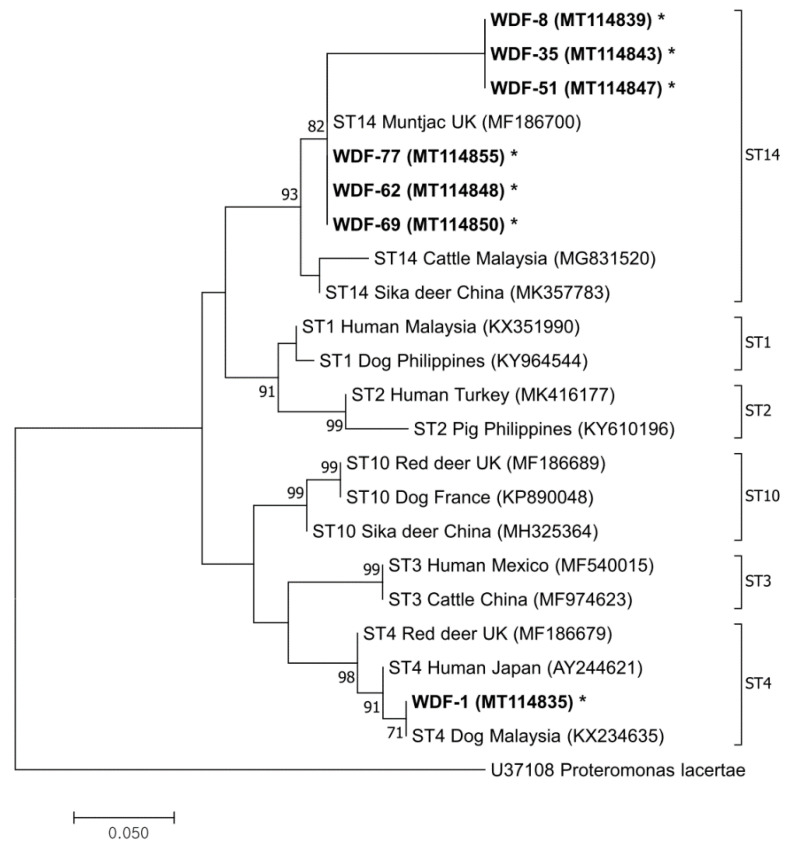
A phylogenetic tree was constructed using the maximum likelihood method based on the Tamura–Nei model (1000 iterations) using the *Blastocystis* 18S rRNA nucleotide sequences generated in this study. Representative sequences identified in this study are marked in bold with asterisks. *Proteromonas lacerate* was used as an out-group. The scale bar means phylogenetic distance.

By conducting a phylogenetic analysis, of 40 representative samples analyzed, 26 sequences were successfully obtained, and, among them, 22 nucleotide sequences were submitted to GenBank as accession numbers MT114835–MT114856, and the remaining four nucleotide sequences were not submitted because they were identical to some of the 22 nucleotide sequences. Selected in a balanced way according to sex, region, and season, we confirmed two subtypes of *Blastocystis* according to the GenBank database: ST4 (*n* = 1) and ST14 (*n* = 25) (Table 2 and Figure 1). ST14 is known to be specifically detected in ruminants including cattle, and ST4 is detected in several hosts, including humans, cattle, and monkeys, so it has zoonotic potential [15]. The ST4 sequence (MT114835) identified in this study had 99.3–99.7% identity with those detected from humans (AY244621 and KX351987). Meanwhile, *Blastocystis* subtypes in Cervidae vary according to species. ST4 and ST10 were found in Eurasian elk and red deer. ST14 was found in Eurasian elk and muntjac deer [16]. ST5, ST10, and ST13 were found in roe deer, fallow deer, and mouse deer, respectively [14].

To summarize, this is the first study on *Blastocystis* in KWD. Among the 125 fecal samples collected throughout Korea, 51 were positive for *Blastocystis* (40.8%) by PCR. Further study on sex is needed to clarify the aforementioned result because the number of samples in this study is inadequate to conclude the correlation between sex and *Blastocystis* infection. By phylogenetic analysis, two subtypes of *Blastocystis* were found in KWD fecal samples: ST4 and ST14 and both subtypes were also detected in other mammals, such as cattle, and ST4 was even found in humans. Because the infection rate of *Blastocystis* in KWD is relatively high and ST4, which was detected in at least one KWD fecal sample, has zoonotic potential, the occurrence of *Blastocystis* infection in KWD cannot be ignored. Therefore, continuous attention is required on the transmission potential of *Blastocystis* between wildlife and domestic animals and humans.

## 3. Materials and Methods

### 3.1. Sample Collection and Ethics

A total of 125 fresh fecal samples were collected throughout Korea from March 2017 to June 2018. When citizens reported that KWD were killed by vehicles on highways or roadsides, government officials visited the sites and transported the corpses to the National Institute of Environmental Research, Ministry of Environment, Korea. Fecal samples were collected from the dead bodies at the institute and then transported to Kyungpook National University College of Veterinary Medicine for molecular analysis. Based on the above description, this study did not require ethical approval since fecal samples were collected from road-killed KWD, and this process did not cause any harm to the animals.

Collection regions were classified into three groups according to provincial boundaries: northern (Gangwon and Gyeonggi), central (Jeonbuk), and southern (Gyeongnam and Gyeongbuk) regions. Data, including sex, region, and collection date, were recorded for data analysis. Samples with unclear information were recorded as “unknown”.

### 3.2. DNA Extraction and PCR

**Figure 2:**
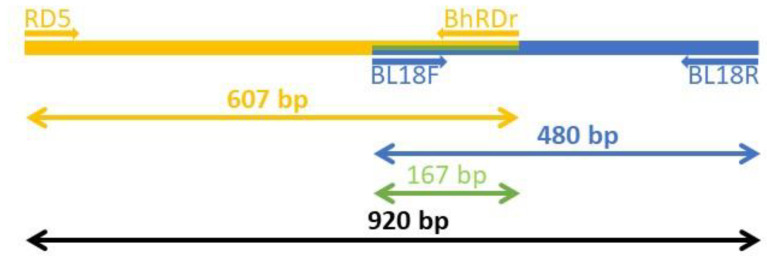
Two different single-round PCRs were used for the detection of *Blastocystis* 18S rRNA fragments that contained overlapping regions. The amplicons using the RD5/BhRDr primers (607 bp) and the amplicons using the BL18F/BL18R primers (480 bp) were designed to overlap in the middle (167 bp).

DNA extraction was performed using the QIAamp^®^ Fast DNA Stool Mini Kit (Qiagen, Hilden, Germany). All DNA extraction procedures were conducted according to the instructions included in the kit. PCR amplification for samples was performed using AccuPower^®^ HotStart PCR Premix (Bioneer, Daejon, Korea). To obtain longer lengths of amplicons, two different single-round PCRs (Figure 2) were used for the detection of *Blastocystis* 18S rRNA fragments with an overlapping region (167 bp). The BL18F (5′ GGAGGTAGTGACAATAAATC 3′) and BL18R (5′ TGCTTTCGCACTTGTTCATC 3′) primer sets were expected to produce an amplicon size of 480 bp [17], and the RD5 (5′ ATCTGGTTGATCCTGCCAGT 3′) and BhRDr (5′ GAGCTTTTTAACTGCAACAACG 3′) primer sets were expected to produce an amplicon size of 607 bp [18,19]. The combination of two PCRs produced an amplicon size of 920 bp. The PCR reaction was performed in a 20 μL solution including the pair of PCR primers (1 μL forward and 1 μL reverse), 15 μL distilled water, and 3 μL template DNA in each tube. Using Mastercycler Pro^®^ (Eppendorf, Hamburg, Germany), PCR amplifications were performed. PCR amplification conditions were as follows: initial denaturation at 95 °C for 5 min, followed by 35 cycles of 95 °C for 30 s, 58 °C for 30 s, and 72 °C for 30 s, and a final extension at 72 °C for 5 min. Then, electrophoresis was performed on 1% agarose gel, and staining was performed using ethidium bromide. Following electrophoresis, PCR-positive samples were sent to Macrogen (Seoul, Korea) for direct nucleotide sequencing.

### 3.3. Nucleotide Sequencing and Phylogeny

Nucleotide sequences were aligned using the Basic Local Alignment Search Tool with other *Blastocystis* sequences which were available from the GenBank database. Phylogenetic analysis was performed using the *Blastocystis* 18S rRNA gene. We used MEGA7 (Pennsylvania State University, University Park, PA, USA) to make a phylogenetic tree.

### 3.4. Statistics

Statistical analysis for distance and maximum likelihood trees was performed using 1000 bootstrap replications. SPSS (version 25.0; IBM, Armonk, NY, USA) along with the χ^2^ test was used for statistical analysis; *p* < 0.05 was used to denote statistical significance.

## Figures and Tables

**Table 1:** Infection of *Blastocystis* in Korean water deer.

Group		No. Tested	No. (%) Positive	χ^2^ (df)	*p*-Value
Sex	Male	55	21 (38.2)	8.564 (2)	0.015
	Female	30	7 (23.3)		
	Unknown	40	23 (57.5)		
Region	Northern	24	10 (41.6)	3.356 (3)	0.346
	Central	46	22 (47.8)		
	Southern	43	13 (30.2)		
	Unknown	12	6 (50)		
Season	Spring	73	32 (43.8)	0.951 (2)	0.682
	Summer	43	15 (34.9)		
	Unknown	9	4 (44.4)		
Total		125	51 (40.8)		

**Table 2:** Distribution of *Blastocystis* subtypes in Korean water deer.

Subtype		ST4 (*n*)	ST14 (*n*)
Sex	Male	0	8
	Female	0	4
	Unknown	1	13
Region	Northern	0	9
	Central	0	2
	Southern	0	12
	Unknown	1	2
Season	Spring	1	12
	Summer	0	12
	Unknown	0	1
Total		1	25
